# Rescue of nonsense mutations by amlexanox in human cells

**DOI:** 10.1186/1750-1172-7-58

**Published:** 2012-08-31

**Authors:** Sara Gonzalez-Hilarion, Terence Beghyn, Jieshuang Jia, Nadège Debreuck, Gonzague Berte, Kamel Mamchaoui, Vincent Mouly, Dieter C Gruenert, Benoit Déprez, Fabrice Lejeune

**Affiliations:** 1 Université Lille Nord de France, IFR142, Lille, France; 2 Inserm, Equipe AVENIR, Lille, France; 3 Institut Pasteur de Lille, Lille, France; 4 INSERM U761 Biostructures and Drug Discovery www.deprezlab.fr, Lille, F-59000, France; 5 Faculté de Pharmacie, Université Lille Nord de France, F-59000, Lille, France; 6 PRIM (www.drugdiscoverylille.org), F-59000, Lille, France; 7 Institut de Myologie, UM76, Paris, France; 8 Université Pierre et Marie Curie, Faculté de Médecine Pierre et Marie Curie, Paris, France; 9 Inserm, UMRS 974, Paris, France; 10 CNRS, UMR, 7215, Paris, France; 11 Departments of Otolaryngology-Head and Neck Surgery and of Laboratory Medicine, Eli and Edythe Broad Center for Regenerative Medicine and Stem Cell Research, Helen Diller Family Comprehensive Cancer Center, Institute for Human Genetics, University of California, San Francisco, San Francisco, CA, USA; 12 Department of Pediatrics, University of Vermont College of Medicine, Burlington, VT, USA; 13 Present address: Unité des Aspergillus, Institut Pasteur, 25 rue du Dr Roux, 75015, Paris, France

**Keywords:** NMD/nonsense mutation/readthrough/RNA/small molecules

## Abstract

**Background:**

Nonsense mutations are at the origin of many cancers and inherited genetic diseases. The consequence of nonsense mutations is often the absence of mutant gene expression due to the activation of an mRNA surveillance mechanism called nonsense-mediated mRNA decay (NMD). Strategies to rescue the expression of nonsense-containing mRNAs have been developed such as NMD inhibition or nonsense mutation readthrough.

**Methods:**

Using a dedicated screening system, we sought molecules capable to block NMD. Additionally, 3 cell lines derived from patient cells and harboring a nonsense mutation were used to study the effect of the selected molecule on the level of nonsense-containing mRNAs and the synthesis of proteins from these mutant mRNAs.

**Results:**

We demonstrate here that amlexanox, a drug used for decades, not only induces an increase in nonsense-containing mRNAs amount in treated cells, but also leads to the synthesis of the full-length protein in an efficient manner. We also demonstrated that these full length proteins are functional.

**Conclusions:**

As a result of this dual activity, amlexanox may be useful as a therapeutic approach for diseases caused by nonsense mutations.

## Background

One third of genetic inherited diseases involve a premature termination codon (PTC) [1]. In most cases, the primary mechanism whereby a nonsense mutation has an effect is through the degradation of that mRNA by a surveillance mechanism called nonsense-mediated mRNA decay (NMD) and not through translation of the mutant mRNA into a truncated protein (for reviews see [2-6]). Several therapeutic strategies have been developed to overcome the presence of nonsense mutations. One approach consists in promoting PTC-containing exon skipping during the RNA splicing process. For example, this approach has been successfully used to eliminate exon 51 mutations of dystrophin gene of patients with Duchenne muscular dystrophy (DMD) [7,8]. The limitations of this approach are: 1) to ensure that the truncated protein is functionally viable, 2) to control the number of exons skipped such that the resultant mRNA is in-frame with an intact open reading frame (ORF), and 3) to reach a sufficiently high level of the skipped exon mRNA to have functional significance.

A second strategy involves the incorporation of a random amino-acid at the PTC position through PTC read-through mechanism. PTC read-through results in the synthesis of a full-length protein that is functional when the PTC is not at a crucial position (i.e. the original amino-acid can be replaced without loss of function). A few molecules have been shown to activate PTC read-through. These include aminoglycoside family members such as G418, or PTC124 (ataluren) [9,10]. However, even in presence of these molecules, the efficiency of PTC read-through is low [9,11]. One reason for this low read-through efficiency is that mutated mRNAs are often substrates for NMD, depleting substrates available for read-through. Therefore, inhibition of NMD may augment PTC read-through [12]. Because NMD occurs upstream of the bulk of translation, regardless of the functional status of the truncated protein, its inhibition may represent an attractive way to treat nonsense-mutation mediated genetic diseases, associated or not to read-through activation [13,14].

Although NMD has been found in eukaryotes from Yeast to Human, the mechanism underlying degradation of PTC-containing mRNAs appears to be species-specific. In mammalian cells, NMD involves 4 main factors: UPF1, UPF2, UPF3 (also called UPF3a) and UPF3X (also called UPF3b). The actual role of UPF proteins remains unclear and certain UPFs are not required for all NMD reactions [15-17]. UPF proteins are recruited to the mRNP in a sequential manner: UPF3 or UPF3X arriving first, then UPF2 and finally UPF1. It is the presence of UPF proteins downstream of a PTC that promotes the activation of NMD on a specific mRNA during the first/pioneer round of translation [18]. As was first demonstrated by tethering any UPF protein to the 3’UTR of β-globin mRNA [19], the presence of UPF proteins downstream of a normal mRNA stop codon activates NMD.

In our attempt to identify NMD inhibitors that could potentially enhance PTC-read-through as new therapies for nonsense mutation-mediated diseases, we previously identified the first NMD-specific inhibitor, NMDI 1 [20]. However, this compound is a new chemical entity based on an indole structure that will require a long and risky optimization and development process before any clinical use. In order to accelerate access to the clinic, we have screened a library of 1200 marketed drugs. We present here evidences that amlexanox stabilizes nonsense mutation containing mRNAs and induces the synthesis of full-length proteins from these mRNAs. Amlexanox might therefore represent a potential new therapeutic molecule to abolish the consequences of a nonsense mutation.

## Methods

### Chemistry

NMR spectra were recorded on a Bruker Avance 300. Chemical shifts are in parts per million (ppm). Mass spectra were recorded with a LCMS (Waters ZQ Micromass). HPLC analyses were performed using a C18 XBridge 3.5 μm particle size column (50 x 4.6 mm). HPLC gradient started from 98% H2O/0.1% formic acid, reaching 98% CH3CN/0.1% formic acid within 5 or 10 min at a flow rate of 2 mL/min. All commercial reagents and solvents were used without further purification. Purification yields were not optimized.

3-(N-Hydroxycarbamimidoyl)-benzoic acid methyl ester.

3.398 mmol of 3-Cyano-benzoic acid (Sigma Aldrich Fluka, 15,716-3, CAS 1877-72-1) were suspended in dichloromethane (10 mL) containing 5% of methanol. A 0.1 equivalent of 4-dimethylaminopyridine and 1.1 equivalent of dicyclohexyl carbodiimide was added at 0°C and stirred for 5 hours. Dicyclohexylurea was then filtered, and the solvent was removed by evaporation. After a second precipitation of dicyclohexylurea in diethylether, filtration and evaporation to dryness, 3-Cyano-benzoic acid methyl ester was obtained as a white powder. The crude residue was then dissolved in absolute ethanol (10 mL) containing a 1.4 equivalent of diisopropylethylamine. Hydroxylamine chlorhydrate (1.3 eq) was added to the mixture, which was refluxed for 4 hours and then ethanol was evaporated to dryness. The residue was dissolved in ethyl acetate and washed with water and brine. The organic layer was dried over magnesium sulfate and evaporated to dryness to yeld 510 mg of a white powder (yield = 77%, Purity LC = 88%)

LC: tR = 1.07 min (5 min), MS (ESI+): m/z = 195.1 [M + H]+

The 3-(N-Hydroxycarbamimidoyl)-benzoic acid methyl ester (2.626 mmol) was suspended in toluene (10 mL) in presence of pyridine (1.1 eq), followed by the addition of 2-Fluoro-benzoyl chloride (Sigma Aldrich Fluka, 120847-25 G, CAS 393-52-2) and 3 hours of reflux. Toluene and pyridine were removed by evaporation. The crude residue was then dissolved in ethyl acetate and washed with aqueous acidic and basic solutions. The organic layer was dried over magnesium sulfate and evaporated to dryness to give 700 mg of a white powder (yield = 89%, Purity LC = 92%).

LC: tR = 3.35 min (on 5 min), MS (ESI+): m/z = 298.91 [M + H]+

The 3-[5-(2-Fluoro-phenyl)- [1,2,4] oxadiazol-3-yl]-benzoic acid methyl ester (2.35 mmol) was suspended in a 1 M NaOH solution in methanol 1 M (10 mL). After 2 hours, the suspension was heated to 50°C and stirred overnight. After 16 hours, the reaction mixture was acidified to pH 4 with 1 M chlorhydric acid. The methanol was evaporated and the product was extracted from aqueous solution with ethyl acetate. The organic layer was then dried over MgSO_4_. The product was crystallized in a mixture 60/40 ethanol/DCM and yielded 245 mg of a white crystal (yield = 36%, purity LC = 97%).

LC: tR = 6.64 min (on 10 min), MS (ESI+): m/z = 284.95 [M + H]+

1 H NMR (CD2Cl2): δ 8.61 (t, J = 1.4 Hz, 1 H), 8.30 (dt, J = 7.8 Hz and J = 1.2 Hz 1 H), 8.24 (td, J = 7.6 Hz and J = 1.7 Hz, 1 H), 8.15 (dt, J = 7.8 Hz and J = 1.4 Hz, 1 H), 7.9 (m, 1 H), 7.72 (t, J = 7.7 Hz, 1 H), 7.55 (d, J = 8.5 Hz, 1 H), 7.49 (q, J = 7.6 Hz, 1 H).

The library of drugs contains more than 1200 pure active pharmaceutical compounds from international pharmacopeias as pure powders. Screening of the compounds was carried out from 96-well plates at 10 μM in DMSO.

### Cell culture and chemical exposure

HeLa cells, expressing firefly luciferase mRNA and one of the fusion protein MS2-UPF, were grown in DMEM supplemented with 10% FBS, 2.5 mg/ml blasticidin and a mixture of 1 U/ml penicillin and 1 mg/ml of streptomycin at 37°C and 5% CO_2_. Calu-3 and Calu-6 cells were grown in RPMI medium supplemented with 10% FBS and a mixture of 1 U/ml penicillin and 1 mg/ml of streptomycin at 37°C and 5% CO_2_. DMD cells were grown in DMEM/199 medium (4:1) supplemented with 20% FBS, 10^-7^ M dexamethasone, 2.5 ng/ml HGF and a mixture of 1 U/ml penicillin and 1 mg/ml streptomycin at 37°C and 5% CO_2_. Cell differentiation was initiated in DMEM supplemented with 10 μg/ml insulin and 100 μg/ml transferrin. 6CFSMEo- cells [21,22] were grown in α-MEM supplemented with 10% FBS, 1 mM L-glutamine and a mixture of 1 U/ml of penicillin and 1 mg/ml of streptomycin at 37°C and 5% CO_2_. Compounds were added 20 hours before harvesting the cells with the exception of the DMD cells which were exposed to the compounds for 48 hours starting with the addition of the differentiation medium.

### Immortalization of DMD cells

*hTERT* and *Cdk4* cDNA were cloned into distinct lentiviral vectors containing respectively the puromycin and neomycin selection markers. Transduction with lentiviral vectors were performed overnight in the presence of Polybrene (4 mg/ml; Sigma-Aldrich). Transduced cell cultures were submitted to selection in the presence of puromycin (0.2 μg/ml) and/or neomycin (0.3 mg/ml) for 8 days. The infected cells were then seeded at clonal density. Clones selected were isolated using glass cylinders and their proliferation and differentiation capacities were characterized in the culture conditions described above. The clone used in this study presented growth and differentiation capacity similar to that observed on the original primary culture.

### Expression constructs

HeLa cells stably expressing firefly luciferase mRNA with MS2 binding sites (bs) in the 3’UTR were obtained by transferring Fluc cDNA with MS2 bs from pCFluc (gift from Dr. Jens Lykke-Andersen) to the pLenti6/V5 using the PCR-based TOPO directional cloning system (Life Technologies) (sense primer: 5’CACCATGGAAGACGCCAAAAACAT3’ and antisense primer: 5’TGACACTATAGAATAGGGCC3’). The lentiviral particles produced with ViraPower Lentiviral Expression System (Life Technologies) were used to transduce the HeLa cells. Stable Fluc expressing clones were selected and isolated using a selective medium containing 2.5μg/ml Blasticidin.

The MS2-UPF expression vectors were constructed by transferring MS2-UPF cDNA from the pMS2-UPF proteins vector (gift from Dr. Jens Lykke-Andersen) into pLenti4/V5-DEST vector using Gateway Cloning according the manufacturer’s instructions. Each MS2-UPF cDNA was cloned using the following primers: sense: 5’GGGGACAAGTTTGTACAAAAAAGCAGGCTCACCATGGCTTCTAACTTTACTCAG3’; and antisense: 5’GGGGACCACTTTGTACAAGAAAGCTGGGTTA\ATTTAGGTGACACTATAGAA3’.

### Screening

HeLa cells stably expressing firefly luciferase were transduced with the recombinant lentiviral constructs containing one of the four pLenti4/V5-MS2-UPF plasmids. Cells were seed into 96 well plates two days after infection and then exposed to the chemical compounds for 20 hours. Plates were loaded into a Tristar LB 941 microplate reader (Berthold) after the addition of Steadylite luciferase substrate (PerkinElmer) in order to measure Luciferase activity in each well for 1 second. Each plate was read 3 times.

### RNA analysis

RNA was purified using RNazol (MRC) according to the manufacturer’s instructions. A portion (1/5) of the RNA preparation was evaluated by reverse-transcription (RT) PCR (RT-PCR) using Superscript II (Life Technologies) for 2 hours at 42°C in presence of random hexamer for RT. The resultant cDNAs were PCR amplified in presence of dCTP( α^33^P) (Perkin Elmer) with primers indicated in Table 1.

**Table 1:** listing of primer sequences used in this study for PCR amplification

**Primers**		**Sequences**
GAPDH	Sense	5’-CATTGACCTCAACTACATGG-3’
	Antisense	5’-GCCATGCCAGTGAGCTTCC-3’
p53	Sense	5’-ATGTGCTCAAGACTGGCGC-3’
	Antisense	5’-GACAGCATCAAATCATCC-3’
Dystrophin	Sense	5’-TCCTGGCATCAGTTACTGTG-3’
	Antisense	5’-CCAGTGGAGGATTATATTCC-3’
CFTR	Sense	5’-GGCCAGAGGGTGGGCCTCTT-3’
	Antisense	5’-CACCCTGTCGGAGGGGCTGA-3’
p21	Sense	5’-CGAGGCACTCAGAGGAG-3’
	Antisense	5’-TCCAGGACTGCAGGCTTCC-3’

PCR products were quantified using Personal Molecular Imager and QuantityOne quantification software (Bio-Rad).

### Glycosylation analysis of CFTR and CFTR immunoprecipitation

Cells were lysed in RIPA buffer (1% deoxycholic acid, 1% Triton x100, 0.1% SDS, 50 mM Tris pH7.4 and 150 mM NaCl) containing HALT protease inhibitor cocktail (Pierce). Then lysats were incubated with 10,000 units of PNGase (New England Biolabs) or 10,000 units of EndoH (New England Biolabs) for 4 hours at 37°C. Cells extracts were then used for the immunoprecipitation of CFTR. Briefly, non specific interactions with protein G agarose were removed by incubating protein extracts with 50 μl of protein G agarose beads (Pierce) for 30 minutes at 4°C. Then protein extracts were incubated with mAb 24–1 antibody for 2 hours at 4°C before to add 50 μl of protein G agarose beads and incubate for an additional 2 hours at 4°C. Beads were finally washed three times with RIPA buffer. Elution of CFTR was obtained by adding 50 μl of 2x sample buffer and vortexing before centrifuging samples and collecting the supernatant.

### Western blot hybridization

Proteins were isolated in the following lysis buffer: 50 mM Tris pH7, 20 mM EDTA and 5% SDS. All protein extracts, with the exception of dystrophin, were analyzed by 10% SDS-PAGE. Dystrophin analysis was carried out using 7.5% SDS-PAGE. After migration, proteins were transferred onto a nitrocellulose membrane and then exposed to the primary antibody. The primary antibodies used were: anti-p53 (D01; Santa-Cruz), anti-dystrophin against an N-terminal epitope (4C7; Santa-Cruz), anti-dystrophin against a C-terminal epitope (exons 77–78) (Abcam), anti-CFTR against an N-terminal epitope (MM13-4; Millipore), anti-tubulin (Epitomics) and anti-phosphorylated eIF2α (Epitomics). The primary antibody incubation is followed by a secondary antibody incubation using anti-mouse or rabbit antibody (Jackson Immuno Research). Proteins finally detected using SuperSignal West Femto Maximum Sensitivity Substrate (Pierce).

### SPQ Halide-efflux Assay

Cells were seeded in 96-well plates and loaded overnight with 10 mM SPQ fluorophore (6-methoxy-N-(3-sulfopropyl)-quinolinium) (Life technologies). Cells were washed twice with I^-^ buffer (135 mM NaI, 2.4 mM K_2_HPO_4_, 0.6 mM KH_2_PO_4_, 1 mM MgSO_4_, 1 mM CaSO_4_, 10 mM dextrose and 10 mM Hepes, pH 7.3) and then incubated in iodide buffer for 30 min. After establishing the basal fluorescence (2 min), the iodide buffer was replaced with NO_3_^-^ buffer (135 mM NaNO_3_ instead of NaI) containing 20 μM forskolin and 200 μM IBMX. Fluorescence was then evaluated for a further 10 min. Fluorescence intensities were measured every 15 seconds using the Tristar LB 941 microplate reader (Berthold) equipped with a 340 nm excitation filter and a 450 nm emission filter.

### Translation efficiency

Calu-6 cells were incubated with DMSO or 25 μM amlexanox for 20 hours before harvesting cells, or with cycloheximide (200 μg/ml) 4 hours before harvesting cells. Newly synthesized proteins were measured using Click-iT AHA for Nascent Protein Synthesis kit (Life Technologies). Briefly, 30 min. before harvesting cells, L-AHA (L-azidohomoalanine) is added to the cell culture medium in order to be incorporated into newly synthesized proteins. Then Cells were washed three times with PBS and lysis buffer was added to the cells. After 30 min. on ice, lysat was centrifuged and supernatant was collected. TAMRA molecule was then bound to the L-AHA modified amino acid using Clik-iT reaction buffer (Life technologies) by following the recommendations of the manufacturer. Finally, TAMRA was detected and quantified by western-blot using a DNR MF-ChemiBIS 3,2 (DNR) and Multi Gauge software (FUJIFILM).

### Cell viability

Cells were harvested after trypsin treatment, centrifuged 3 min at 200 g and resuspended in PBS. Tali dead cell red (propidium iodide) reagent was then added to the cells. After a 5 min incubation in the dark, the cells were loaded into Tali image-based cytometer (Life Technologies).

### Immunofluorescence

DMD cells were grown on ibiTreat surface μ-dishes (Ibidi) and differentiated as described above. Cells were fixed 10 min in 95% ethanol at room temperature 48 hours after initiating differentiation. The fixed cells were washed twice in PBS and incubated with the C-terminal anti-dystrophin antibody for 1 hour at room temperature, washed twice with PBS and incubated with the secondary anti-rabbit antibody conjugated with Alexa 488 fluorophore (Life Technologies) for 1 hour at room temperature. The cells were then incubated with Hoechst stain for 1 min at room temperature before adding Vectashield (ABCYS) mounting medium and sealing a coverslip.

## Results and discussion

### Identification of amlexanox as an NMD inhibitor

**Figure 1:**
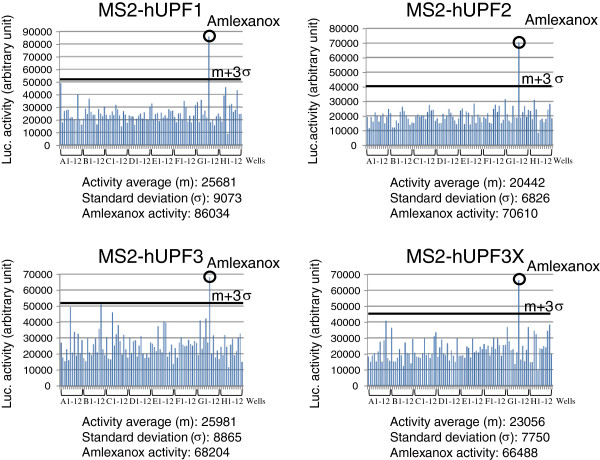
**Identification of amlexanox as putative NMD inhibitor.** Luciferase activity was measured from cells expressing the Firefly luciferase with MS2 binding sites in its 3’UTR and MS2-UPF1 (up and left), MS2-UPF2 (up and right), MS2-UPF3 (down and left) or MS2-UPF3X (down and right). Tested drugs were added in columns 2 to 11 of each 96-well plate, the vehicle (DMSO) was added in positions A1, B1 and C1. No compounds were added in the rest of column 1 and column 12 of each plate, as negative controls. The luciferase activity is provided in arbitrary units. The mean luminescence values were calculated from all test compounds and for each plate. The threshold of hit selection was calculated for each plate and is represented by the black thick line: m + 3σ (mean plus 3 standard deviations).

A tethering-based screening system [19] has been developed to identify NMD inhibitors from chemical libraries. Briefly, firefly luciferase (Fluc) mRNA was stabilized when the function of hUPF1, hUPF2, hUPF3 or hUPF3X was blocked by a chemical molecule. The assay relies on the transfection of a cell line that stably expresses a Fluc mRNA with 6 MS2-binding sites in the 3’UTR, with vectors that express MS2 fusion protein with one of the four hUPF proteins. Cells were incubated in 96 well-plates with the test compounds. Luciferase activity after adding a luciferase substrate mixed with a lysis buffer was measured three times. All drugs have been tested once. The hit selection threshold is the mean plus 3 standard deviation. Hit rates were 0.44%; 0.18%; 0.18% and 0.36% in UPF1; 2; 3 and 3X respectively. Amlexanox (Sequoia Research Products, UK) is the only one drug confirmed on the primary assay after screening. It significantly increases the luciferase activity in the four cell lines expressing one of the MS2-UPF proteins, suggesting that this compound might block NMD after recruitment of hUPF1, which is the most downstream factor among the four tested (Figure 1 and Additional file 1: Tables S1-4 for tables of luciferase measures).

To further characterize amlexanox, we determined whether it is able to increase the amount of endogenous nonsense mutation-containing mRNAs in cell lines issued from patients suffering from nonsense-mutation-mediated lung cancer, Duchenne muscular dystrophy (DMD) or cystic fibrosis (CF). In these cells the mutated mRNAs code for truncated p53, dystrophin and the CF transmembrane conductance regulator (CFTR), respectively. Interestingly, with these cell lines, it was possible to measure the effect of our compound on the three possible nonsense codons located in three different nucleotide environments.

**Figure 2:**
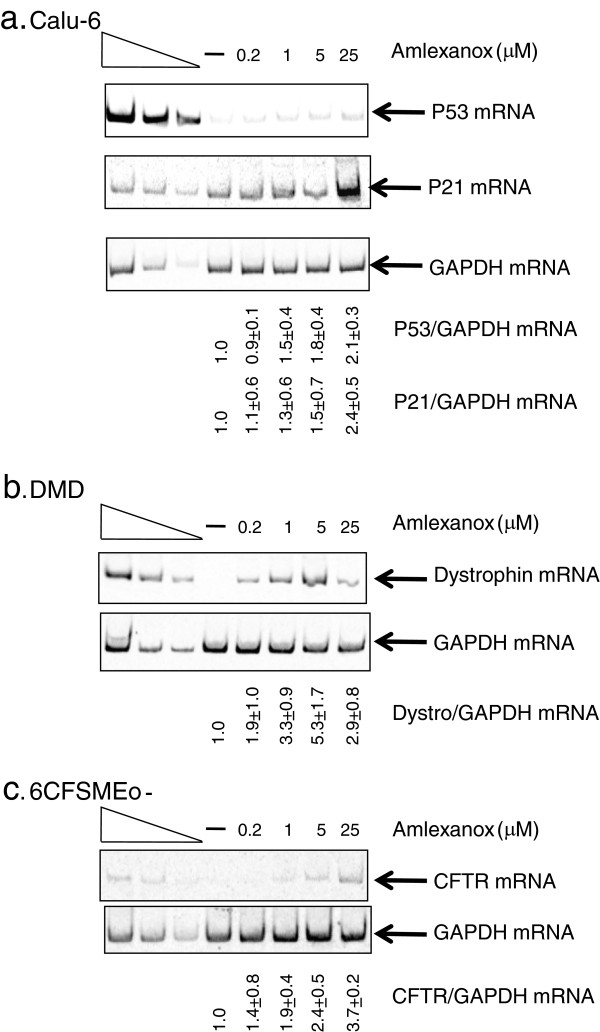
**Amlexanox increases the amount of nonsense mutation-containing mRNAs.** Increasing amounts of amlexanox are added to the cell culture medium of Calu-6 cells (**A**), DMD cells (**B**) or 6CFSMEo- cells (**C**). RNAs are purified, reverse transcribed and PCR is performed to measure the level of p53, dystrophin or CFTR mRNA, respectively. GAPDH level is used to normalize the amount of the nonsense mutation-containing mRNAs level. The three left lanes represent a two-fold serial dilution of RNA from untreated cells (Calu-3 cells for (A) and (C) or WT myoblasts for (B)). Quantifications are given as a ratio of P53 or P21 mRNA on GAPDH mRNA level and normalized to DMSO treatment which is given as 1. Quantifications are based on at least 3 independent experiments and the average quantification is indicated under each gel with standard deviation.

**Figure 3:**
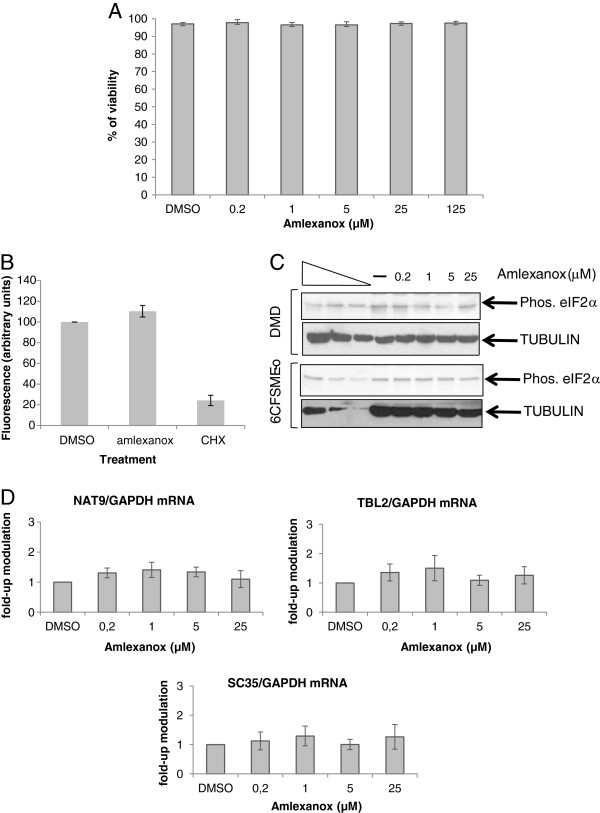
**Amlexanox is not toxic, does not inhibit general translation and does not affect natural NMD substrates expression.** (**A**) Calu-6 Cell viability was measured after 20 hours of cell incubation with increasing amounts of amlexanox or with DMSO. These results combine three independent experiments. (**B**) Measure of translation efficiency after amlexanox treatment. Calu-6 Cells were incubated with DMSO, amlexanox or cycloheximide (CHX) before to measure translation efficiency using Click-iT AHA for nascent protein synthesis kit (Life technologies). This result is representative of 2 independent experiments. (**C**) Western-blot showing that the level of phosphorylated (Phos.) eIF2α does not increase in the presence of amlexanox in DMD cells (upper panels) or 6CFSMEo- cells (lower panels). The three left lanes represent a two-fold serial dilution of cell extract from untreated WT myoblasts (upper panels) or Calu-3 cells (lower panels). (**D**) The level of three NMD natural target mRNAs was measured from Calu-6 cells as described in Materials and Methods from three independent experiments.

The lung cancer cell line, Calu-6 (ATCC, USA) has a homozygous TGG→TGA mutation at codon 196 of p53. These cells were incubated with increasing concentrations of amlexanox ranging from 0.2 to 25 μM for 20 hours or with DMSO as a control (Figure 2A). Reverse transcribed RNAs from Calu-3 cells (ATCC, USA) were used as a reference. Calu-3 cells are derived from a lung adenocarcinoma and they express both p53 and CFTR at relatively high level (RNA and protein). A greater than 2-fold increase in the p53 mRNA amount was observed at 25 μM of amlexanox in Calu-6 cells suggesting an inhibition of NMD. At higher concentrations, a change in the cell morphology was observed, suggesting that amlexanox concentrations higher than 25 μM might interfere with cellular metabolism. However, the cell viability even at 125 μM was comparable to that observed with the DMSO alone (Figure 3A). Therefore, to avoid any confounding results, 25 μM was the highest concentration used. A faint band corresponding to p53 mRNA was detected in the DMSO sample. This residual expression of p53 in Calu-6 cells has already been observed previously [23] and could represent a subpopulation of NMD-protected p53 mRNAs (such as nuclear p53 mRNAs) or cytoplasmic untranslated p53 mRNAs.

We then tested an immortalized myocyte cell line from a DMD patient with nonsense mutation (TCT→TAA) in exon 71 at codon 3420 of the dystrophin gene (see Methods). These cells were treated with amlexanox for 48 hours while they were exposed to conditions that promote differentiation and dystrophin expression. As with the Calu-6 cells, increasing concentrations of amlexanox resulted in increased levels (up to 5-fold) of the mutant mRNA (Figure 2B).

The third cell line used (6CFSMEo- cells) is an immortalized cystic fibrosis (CF) airway epithelial cell line derived from a population enriched for submucosal gland epithelial cells [21,22]. These cells are from a patient who is compound heterozygous: one allele is a CTT deletion spanning codons 507 and 508 of CFTR that results in the in frame deletion of a phenyalanine (ΔF508), the other allele is a CAG→TAG nonsense mutation at the codon 2 (Q2X) of the CFTR gene [21]. A previous study indicated that there was little or no expression of the ΔF508 nor of the Q2X alleles in the 6CFSMEo- cells [22]. When the cells were treated with increasing concentrations of amlexanox a 3- to 4-fold increase in the CFTR mRNA amount was detected at 25 μM of amlexanox (Figure 2C). Even though we favor the hypothesis that amlexanox stabilized PTC-containing mRNAs, we cannot exclude the possibility that amlexanox activated the expression of the ΔF508 CFTR allele. As with Calu-6 cells (p53 PTC-containing mRNA), 25 μM was the most effective for facilitating CFTR mRNA increase in 6CFSMEo- cells (Figures 2A,C). Altogether results from Figures 1 and 2 confirm that molecule amlexanox blocks NMD in various PTCs and PTC environments.

### Cytotoxicity, translation efficiency and stabilization of natural NMD targets

The specificity and cytotoxicity of the amlexanox were evaluated on Calu-6 cells. The effect of amlexanox on cell survival was evaluated after a 20 hours treatment at the working concentrations (0.2 to 25 μM) or at 125 μM of amlexanox. The viability after amlexanox or DMSO incubation was between 95 and 98% (Figure 3A), indicating that amlexanox is not toxic for cells even at 125 μM in the conditions described above.

Inhibition of translation by amlexanox was assayed by incorporating an L-AHA (L-azidohomoalanine) modified amino-acid in newly synthesized proteins. L-AHA is then detected and measured using Click-iT AHA new protein synthesis kit (Life Technologies) (See Methods). Results of Figure 3B show that amlexanox has no significant effect on the level of fluorescence, in contrast to cycloheximide which strongly reduces it, suggesting that the efficiency of translation is not altered by amlexanox.

In addition, the phosphorylation status of eIF2α after amlexanox treatment was assessed since the induction of eIF2α phosphorylation as a function of inhibiting NMD has been demonstrated [24,25] and that would impair translation process. The expression of eIF2α was analyzed in both the DMD and 6CFSMEo- cells with an antibody specific for the phosphorylated isoform of eIF2α (Figure 3C). No increase in the eIF2α phosphorylation was observed, suggesting that NMD inhibition does not always require an induction of eIF2α phosphorylation.

The effect of amlexanox-mediated-NMD inhibition was also assessed on genes that normally use the NMD pathway to regulate their expression. The mRNA levels of three genes that are natural NMD targets (Nat9, Tbl2 and SC35) [26] were assayed in Calu-6 cells (Figure 3D). For each of these targets, we measured an average of 1.2 -fold increase in mRNA level and no dose-related effect. This suggests that amlexanox does not affect the regulation of these (and possibly all) natural targets of NMD and also suggests that the increase observed in nonsense-containing mRNAs is not simply due to a general transcriptional up-regulation by amlexanox.

### Amlexanox induces protein synthesis from nonsense mutation-containing mRNA

**Figure 4:**
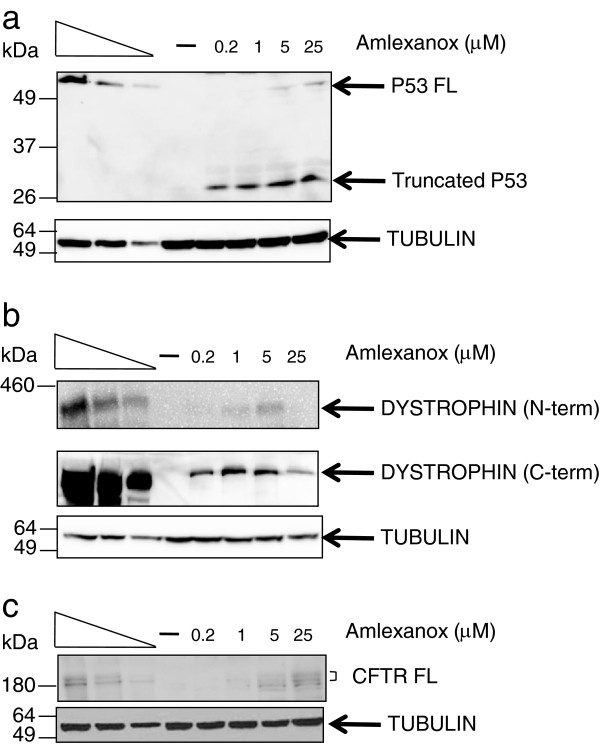
**Amlexanox treatment leads to the synthesis of truncated and/or full length proteins from nonsense mutation-containing mRNAs.** Calu-6 **(A)**, DMD **(B)** or 6CFSMEo- **(C)** cells were incubated with amlexanox molecule or with DMSO (−) as a control before purifying proteins and performing Western blot to detect p53, dystrophin or CFTR protein, respectively, using antibodies raised against N-terminal part of the proteins (and C-terminal part of the dystrophin protein as mentioned). The three left lanes represent a two-fold serial dilution of cell extract from untreated cells (Calu-3 cells for **(A)** and **(C)** or WT myoblasts for **(B)**). Each gel is representative of at least 2 independent experiments. On the left of each gel is indicated the molecular weight ladder (BenchMark pre-stained for A and C, or HiMark pre-stained for B (Life Technologies)).

Protein synthesis from nonsense mutation mRNAs was analyzed after amlexanox inhibition of NMD. All three cell lines with the PTCs described above were assayed by Western-blot for the presence of truncated p53, dystrophin or CFTR protein. An N-terminal antibody was initially used to detect truncated as well as full-length proteins (Figure 4). After amlexanox treatment, a truncated p53 protein was well detected in Calu-6 cells. This truncated protein was not present in DMSO treated Calu-6 cells or in whole-cell extracts from Calu-3 cells. In addition, a very low level of full-length p53 was also detected when cells were treated with 1 to 25 μM amlexanox. In Calu-6 cells, it appears that the PTC-containing P53 mRNA stabilized by amlexanox can be translated into proteins. These results also suggest that amlexanox can directly or indirectly elicit PTC-readthrough on this particular PTC.

Previous studies clearly demonstrated that PTC-readthrough depends on the identity of the PTC and its nucleotide context [11,27-29]. To determine whether the dystrophin and CFTR PTCs resulted in full-length and/or truncated protein, the two other cell lines were also treated with amlexanox. Increasing concentrations of amlexanox in the culture medium of DMD cells during differentiation induced the synthesis of dystrophin protein with maximum synthesis at a concentration of 5 μM amlexanox, as detected with an anti-N-term antibody (Figure 4B, upper gel). This result was consistent with the observation that maximal dystrophin mRNA stabilization also occurs at 5 μM (Figure 2B). However, it was difficult to distinguish the full length from the truncated form of dystrophin with this antibody because of their high and similar molecular weights (427 vs 400 kDa). Therefore, a C-terminal anti-dystrophin antibody (with an epitope in exons 77–78 of dystrophin so downstream of the PTC position) was used. This antibody also detected dystrophin produced upon amlexanox treatment indicating that at least a portion of the dystrophin synthesized in the presence of amlexanox is the full-length protein (Figure 4B middle gel).

Analysis of CFTR protein expression in 6CFSMEo- cells after treatment with increasing concentrations of amlexanox was a bit more challenging, since the truncated CFTR protein with a stop at the second codon would be purely theoretical [22]. As with the other two cell lines, bands were detected that were consistent with the full-length wild-type CFTR protein produced in Calu-3 cells (Figure 4C).

Interestingly, CFTR is subject to 2 steps of glycosylation occurring first in the endoplasmic reticulum to give the core glycosylated band B CFTR, and then in the Golgi to give the fully mature band C CFTR. To assess the glycosylation level of the CFTR protein isoform obtained after amlexanox treatment, we incubated protein extracts from Calu-3 or 6CFSMEo- cells treated or not with amlexanox with PNGaseF or endoH glycosydases before to immunoprecipate CFTR (Additional file 2: Figure S1). The resulting CFTR protein after amlexanox treatment in 6CFSMEo- cells is processed like the wild-type CFTR from Calu-3 cells with a band C showing some sensitivity to PNGaseF and resistance to endoH. Band B is sensitive to both glycosidases but this isoform was not observed in 6CFSMEo- cells after amlexanox treatment likely due to the low amount of synthesized CFTR.

Figure 4 shows that full-length proteins are synthesized after amlexanox treatment from mRNAs containing nonsense mutations. This strongly suggests that amlexanox promotes PTC-readthrough. However, it was not possible to determine whether amlexanox was equally efficient on any PTC, because the appropriate cell models were not available. For example Calu-3 cells over-express CFTR and synthesize relatively high levels of p53 protein. In the case of DMD cells, both cell lines come from different patients and cannot be effectively compared, because inter-individual variation in the expression of the same gene [30]. However, it does appear that amlexanox induces, at low micromolar concentrations, the synthesis of full-length proteins from any PTC at a level detectable by Western blot.

### Functional analysis of proteins generated after amlexanox treatment

**Figure 5:**
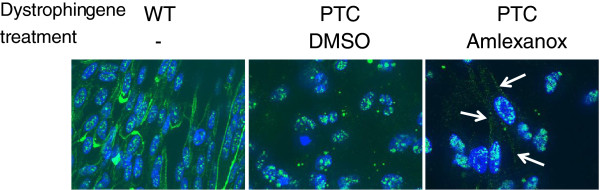
**Dystrophin is found at the cell membrane of DMD cells after amlexanox treatment.** WT (left panel) or DMD cells (two right panels) were incubated in differentiation medium in presence of DMSO or 5 μM of amlexanox for 48 hours. Then cells were fixed and nuclei (blue) were stained using Hoechst reagent and dystrophin (green) was localized using anti-dystrophin raised against the C-terminal part of the protein. White arrows indicate dystrophin localization at the cellular membrane. Scale bar represents 10 μm.

Truncated dystrophin from our DMD cell line would be as functional as the wild-type dystrophin [31]. Therefore, we analyzed the cellular localization of dystrophin in this cellular model. Dystrophin immunolocalization using the antibody that recognizes an epitope in exons 77–78 was distinctly different when comparing WT cells to DMD cells (Figure 5). An intense fluorescent signal (green) was observed under the cytoplasmic membrane of WT cells, but not in the DMD cells treated with DMSO where there appears to be some weak non specific staining. Additionally, DMD cells keep a small size under differentiation conditions unlike WT cells or DMD cells after amlexanox treatment. Indeed, about 20% of cells (n = 329) show a large size representing cells that are in the process of differentiate into myotubes. In presence of amlexanox, dystrophin staining becomes more apparent and can be easily detected at the plasma membrane of 75% of large cells (n = 76), in a pattern similar to what is observed with the wild-type DMD cells (Figure 5, right panel). Thus, these images are consistent with the Western blot analysis, and confirm that full-length dystrophin is synthesized after amlexanox treatment.

**Figure 6:**
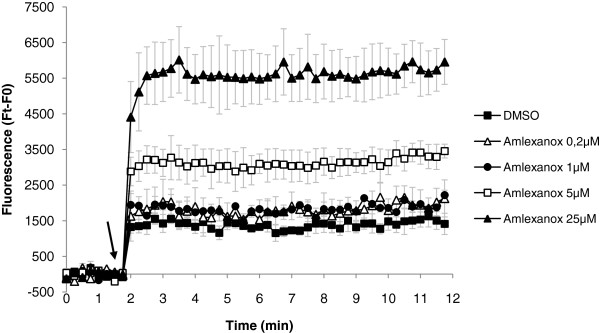
**Amlexanox treatment of 6CFSMEo- cells leads to the synthesis of functional CFTR.** 6CFSMEo- cells were loaded with the halide-sensitive fluorophore SPQ and treated with DMSO (■), 0.2 μM amlexanox (Δ), 1 μM amlexanox (●), 5 μM amlexanox (**□**) or 25 μM amlexanox (▴) for 20 hours. At time (t) = 2 min cAMP-stimulating cocktail was added (arrow). The increase in iodide efflux is shown as mean ± SEM from at least four independent experiments. Ft represents the measure of the fluorescence at the reading time; F0 is an average fluorescence before addition of cAMP agonists.

Since the Calu-6 and 6CFSMEo- cell lines encode a nonfunctional truncated p53 or CFTR proteins, respectively, it was interesting to determine whether a treatment with amlexanox actually rescues the lost functions in these cells. The function of the full-length p53 protein in the Calu-6 cells was determined by measuring the level of p21 mRNA (a transcriptional target of p53 [32]). The rationale for this analysis was that amlexanox-induced increases in functional p53 should result in an increase in the amount of p21 mRNA. The level of p21 mRNA was correlated with the increase in p53 mRNA (at 5 and 25 μM in particular) (Figure 2A). The function of CFTR in 6CFSMEo- cells was evaluated by measuring cAMP-dependent halide efflux as a function of SPQ fluorescence [33]. Cells were loaded with SPQ and incubated with increasing amounts of amlexanox or with DMSO alone. The medium was sequentially switched to 1) an iodide solution to quench the SPQ fluorescence, and 2) to a nitrate solution containing forskolin and IBMX to increase intracellular cAMP levels and activate the CFTR. If CFTR is functional, iodide will be secreted from cells after cAMP stimulation and replaced by nitrate on the anion binding site of SPQ. Unlike iodide, nitrate does not quench the fluorescence of SPQ. Therefore the presence of functional CFTR will be assessed by a rapid increase in the SPQ fluorescence. A dose-dependent in iodide efflux with a maximum effect at 25 μM amlexanox was clearly observed in 6CFSMEo- (Figure 6). Although, a direct comparison cannot be made between 6CFSMEo- cells and Calu-3 cells due to the fact that the latter overexpress CFTR, a set of measures of CFTR functionality in Calu-3 cells using the same SPQ assay is presented in supplemental Additional file 3: Figure S2 and shows that the CFTR activity that we measured in 6CFSMEo- cells after amlexanox treatment is about 20% of the CFTR activity that is measured in Calu-3 cells. Overall, these results demonstrate that amlexanox induces the synthesis of functional CFTR protein in 6CFSMEo- cells.

### Comparison of amlexanox with PTC-readthrough molecules

**Figure 7:**
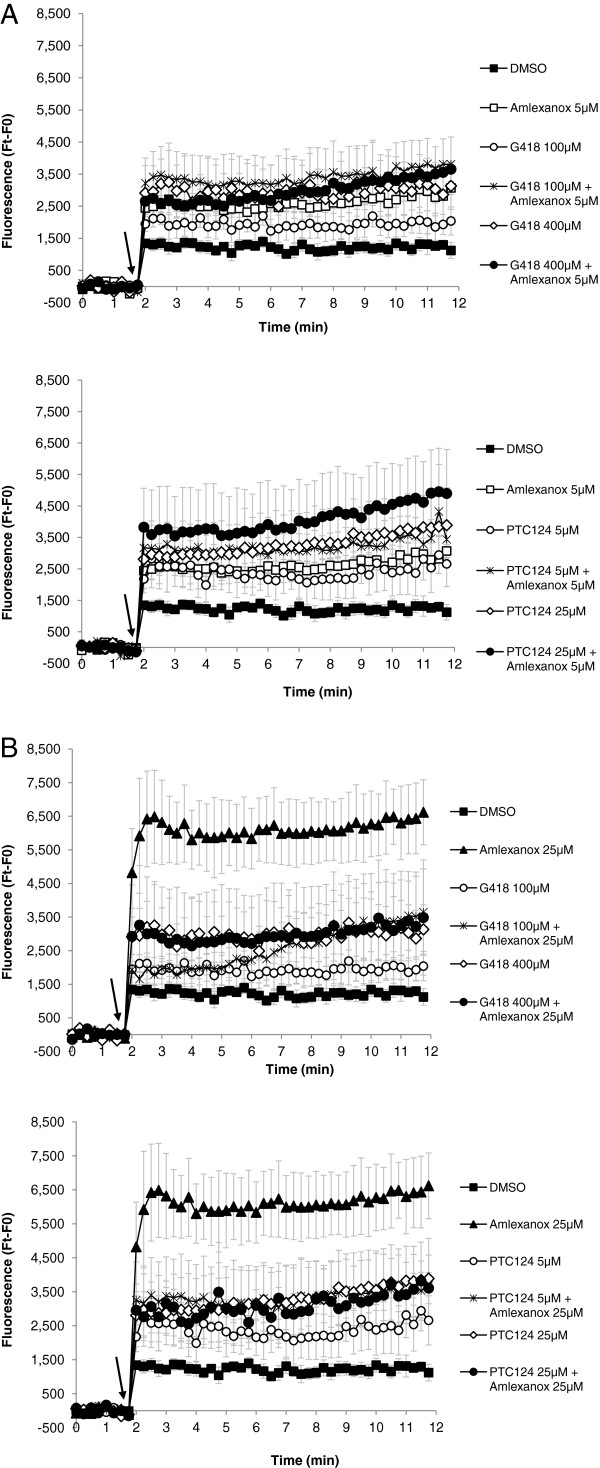
**Comparison of the efficiency of amlexanox alone or in combination with PTC-readthrough molecules. **6CFSMEo- cells were incubated with 5 μM (**A**) or 25 μM (**B**) of amlexanox alone or with G418 (upper panel) or PTC124 (lower panel). At time (t) = 2 min cAMP-stimulating cocktail was added (arrow). The increase in iodide efflux is shown as mean ± SEM from at least four independent experiments. Ft represents the measure of the fluorescence at the reading time; F0 is an average fluorescence before addition of cAMP agonists.

Since amlexanox was able to induce the synthesis of full-length proteins from nonsense mutation-containing mRNAs, it was interesting to compare its efficacy to that of G418 or PTC124, which have already been shown to facilitate PTC-readthrough [9,34,35]. The effect of each molecule and their combinations was evaluated quantitatively by measuring iodide efflux across the plasma membrane. Amlexanox, G418 or PTC124 (for synthesis of PTC124 see Methods) were added to 6CFSMEo- cells for 20 hours either alone or in combinations (Figure 7). Amlexanox was used at 5 μM (suboptimal) and 25 μM (optimal) and G418 was used at 100 and 400 μM, concentrations used previously to show G418 readthrough [11,36,37]. PTC124 was used at 5 and 25 μM, which were previously shown to be optimal for readthrough [9]. First of all, a significant increase in the export of iodide was observed for the 3 components, compared to treatment with DMSO and amlexanox at 5 μM is as potent as G418 at 400 μM or PTC124 at 5 μM (Figure 7A). Although combinations behave with similar efficacy, the combination of amlexanox (5 μM) and PTC124 (25 μM) seems to be slightly more efficient than each molecule alone (Figure 7A lower panel). Interestingly, amlexanox at 25 μM was at least 3 times more effective than any of the single other molecules or combinations (Figure 7B), an important result which could be due to the combined function of amlexanox in both NMD and readthrough processes.

## Conclusions

The studies presented here outlined the features of a new NMD inhibitor, amlexanox (Figure 1). It is probable that amlexanox inhibits NMD at a late stage, since its effect is not abolished by tethering the MS2-UPF1 protein in the 3’UTR of the Fluc mRNA (Figure 1). Amlexanox is shown to stabilize the three different nonsense mutation-containing mRNAs from three cellular human disease models (Figure 2). The work presented here also demonstrates that it is possible to inhibit the degradation of nonsense mutation containing mRNAs without dramatically altering cellular metabolism. Both cell viability (Figure 3A) and translation are not affected by amlexanox treatment (Figure 3B). The observed lack of toxicity of amlexanox could be explained by the redundancy of mRNA level regulation. In our study, the levels of natural NMD substrates are not up-regulated after treatment with amlexanox (Figure 3D), a point that can be explained by the fact that genes using NMD to regulate their expression often use several regulatory mechanisms. For example, SR protein genes use NMD to regulate their expression [38]. The regulation of SR protein gene expression also involves transcription, splicing, translation and/or post-translational events to maintain a functional level of SR proteins [39,40]. These are likely backup regulatory mechanisms to compensate for a lack of response in a specific regulatory pathway. Furthermore, an unusual event must occur to involve NMD regulation as with the SR proteins that employs an NMD mechanism that reduces the amount of translated mRNA and is activated only when the level of a specific SR protein becomes abnormally high [38,41]. These previous studies and the present studies suggest that robustness of gene regulation allows some inhibition of NMD without significantly affecting the profile of gene expression (Figure 3D).

Interestingly, amlexanox not only increases the amount of nonsense mutation-containing mRNAs, but also induces the synthesis of truncated and/or full-length proteins from PTC-containing mRNAs, suggesting activation of PTC-readthrough (Figure 4). This ability to both stabilize nonsense mutation-containing mRNA and promote PTC readthrough has been previously described for G418 [34]. G418 is an aminoglycoside analog, which unfavorable therapeutic index makes it unsuitable for medical applications. Amlexanox is effective at concentrations (5 or 25 μM) that are generally lower than those required for aminoglycosides. We have observed with amlexanox some variations in the sensitivity of cells to treatment which would be due to the nature of the nonsense mutation, the nucleotide environment, and the nature of mRNA or the cell type. Therefore, each cell model with a nonsense mutation needs to be independently assayed to determine the most effective amlexanox working concentration. In our study, The DMD cell model was the most sensitive to amlexanox (Figures 2 and 4). However, DMD cells are distinct from the other cell systems studied, since they require cellular differentiation to activate expression of nonsense-mutation containing dystrophin mRNA. Furthermore, the decrease of the efficacy of amlexanox at 25 μM when compared to effect at 5 μM may be due to a cellular defense that inhibits the entrance of amlexanox into the cells when a specific concentration is reached. We cannot exclude some cellular toxicity at the highest concentration that we didn't detect in Figure 3A due to the difference of cellular model. Alternatively, amlexanox could affect muscle differentiation process or dystrophin expression.

The ability of amlexanox to inhibit NMD and facilitate PTC-readthrough of all the PTCs tested suggests that its mode of action is different from molecules that only activate PTC-readthrough and for which the efficacy is strongly influenced by the identity of the PTC and its nucleotide environment [11].

Recent studies have shown that protein synthesis occurs during the pioneer round of translation [42]. Amlexanox could cause nonsense-mutation read-through during this round of translation, if NMD inhibition and readthrough are concomitant. If readthrough occurs after the pioneer round, it would suggest that NMD inhibition and read-through promotion are two distinct molecular intervention of amlexanox. Clearly, further analysis of the mode of action of amlexanox will be important for its development as a novel therapy for nonsense mutation mediated diseases. These studies may also provide a better understanding of NMD mechanism. While amlexanox appears to be the most potent molecule of the three tested (Figures 6 and 7), further studies using other quantifiable PTC-readthrough systems will be required to determine whether this is a specific or a general property of amlexanox. Indeed, PTC124 has been shown to be efficacious for some nonsense-mutations [9,43] and totally ineffective with others [44,45].

Amlexanox has anti-allergic [46,47] and anti-inflammatory properties [48,49]. It has been used for more than 30 years per os or topically to treat asthma and aphthous ulcers [46,50]. Together with its activity on nonsense mutation containing genes, the relative safety of amlexanox and its current use as an oral treatment of asthma, encourage us to investigate in details its therapeutic potential in other pulmonary diseases such as nonsense mutation mediated cystic fibrosis but not only. To our opinion, amlexanox appears to be a reasonable candidate for a novel therapy to treat disease states that are the result of nonsense mutations. Along with PTC124, amlexanox provides another opportunity for the development of a disease modifying, personalized therapy against several genetic diseases.

## Abbreviations

BS: binding site; CF: cystic fibrosis; CFTR: cystic fibrosis transmembrane conductance regulator; DMD: Duchenne muscular dystrophy; Fluc: firefly luciferase; Min: minute; NMD: nonsense-mediated mRNA decay; ORF: open reading frame; PCR: polymerase chain reaction; Phos.: phosphorylated; PPM: parts per million; PTC: premature termination codon; RT: reverse transcription; UTR: untranslated region.

## Competing interests

Authors declare that they have no competing interests.

## Authors’ contribution

SGH, TB, JJ, ND, KM and FL performed experiments. SGH, TB, GB, VM, DCG, BD and FL interpreted results. SGH, TB, VM, DCG, BD and FL wrote the paper. All authors read and approved the final manuscript.

## Supplementary Material

Additional file 1 **Tables S1 to S4.** measures of the luciferase activity obtained with the plate presented Figure 1 for the 4 MS2-UPF strains. For each strain, the plate was read 3 times by the luminometer (reading 1 to 3) in order to control the luminometer variability. It is an average of these 3 readings that is presented Figure 1. Click here for file

Additional file 2 **Figure S1.** Analysis of the glycosylation status of CFTR in 6CFSMEo- treated with DMSO or 25 μM of amlexanox for 24 hours and Calu-3 cells. 1/10 of CFTR immunoprecipitation from Calu-3 cells and 1/2 of CFTR immunoprecipitation from 6CFSMEo- cells were analyzed by western-blot. Molecular weight marker is indicated on the left side of the gel. Band B (B) and band C (C) are indicated on the right side of the gel. This analysis is representative of three independent experiments. Click here for file

Additional file 3 **Figure S2.** Measure of iodide transport through Calu-3 cell membrane using halide-sensitive fluorophore SPQ assay. At time (t) = 2 min cAMP-stimulating cocktail was added (arrow). The increase in iodide efflux is shown as mean ± SEM from at least four independent experiments. Ft represents the measure of the fluorescence at the reading time; F0 is an average fluorescence before addition of cAMP agonists. Click here for file
