# Supplementary material for: Rescue of nonsense mutations by amlexanox in human cells
Source: Orphanet J Rare Dis. 2012 Aug 31;7:58. doi: 10.1186/1750-1172-7-58 (PMC3562214; doi:10.1186/1750-1172-7-58)
Supplement: Additional file 1 — Tables S1 to S4. measures of the luciferase activity obtained with the plate presented Figure 1 for the 4 MS2-UPF strains. For each strain, the plate was read 3 times by the luminometer (reading 1 to 3) in order to control the luminometer variability. It is an average of these 3 readings that is presented Figure 1. [file 1750-1172-7-58-S1.pdf]

well	reading 1	reading 2	reading 3		well	average readings 1-3
A01	18377	18324	18647		A01	18449.3333
A02	14590	15288	15436		A02	15104.6667
A03	18813	18918	19145		A03	18958.6667
A04	19921	20488	20890		A04	20433
A05	15384	15235	15480		A05	15366.3333
A06	20654	21893	22138		A06	21561.6667
A07	26954	27670	28499		A07	27707.6667
A08	14703	15279	15375		A08	15119
A09	27504	29031	29040		A09	28525
A10	39886	40863	41867		A10	40872
A11	16693	17469	17801		A11	17321
A12	14939	15750	15995		A12	15561.3333
B01	35471	36221	37478		B01	36390
B02	15043	15349	15715		B02	15369
B03	14913	15035	15689		B03	15212.3333
B04	17495	17714	18019		B04	17742.6667
B05	22460	23246	23351		B05	23019
B06	18673	18848	19703		B06	19074.6667
B07	12321	12426	12766		B07	12504.3333
B08	27338	27286	27260		B08	27294.6667
B09	20323	20576	20864		B09	20587.6667
B10	20113	20314	20349		B10	20258.6667
B11	13691	14119	14502		B11	14104
B12	27975	29214	30959		B12	29382.6667
C01	19939	20628	21344		C01	20637
C02	18045	18246	18839		C02	18376.6667
C03	17661	18036	18272		C03	17989.6667
C04	17085	17260	17975		C04	17440
C05	20157	20227	21003		C05	20462.3333
C06	29083	29642	30261		C06	29662
C07	18830	19136	19590		C07	19185.3333
C08	20000	20541	21169		C08	20570
C09	16710	17251	18028		C09	17329.6667
C10	19180	19817	20698		C10	19898.3333
C11	30462	30872	31658		C11	30997.3333
C12	32085	34127	34895		C12	33702.3333
D01	17766	18124	19005		D01	18298.3333
D02	23516	24005	24790		D02	24103.6667
D03	27652	28874	30994		D03	29173.3333
D04	19913	20157	20698		D04	20256
D05	18857	19014	19555		D05	19142
D06	26021	26597	27844		D06	26820.6667
D07	15384	15689	16099		D07	15724
D08	21710	22312	22670		D08	22230.6667
D09	18150	18612	19450		D09	18737.3333
D10	14869	14721	15201		D10	14930.3333
D11	28246	29267	31448		D11	29653.6667
D12	18289	18464	19267		D12	18673.3333

E01	18822	18586	18918	E01	18775.3333
E02	18385	19014	19947	E02	19115.3333
E03	17016	17748	18473	E03	17745.6667
E04	23944	24511	25785	E04	24746.6667
E05	23202	23577	24598	E05	23792.3333
E06	17679	17783	18289	E06	17917
E07	29110	29424	30165	E07	29566.3333
E08	20296	20907	21815	E08	21006
E09	21501	21946	22661	E09	22036
E10	20427	20864	21457	E10	20916
E11	23324	24782	25515	E11	24540.3333
E12	22827	22844	23455	E12	23042
F01	26483	25829	26230	F01	26180.6667
F02	22652	22757	23464	F02	22957.6667
F03	25078	25017	25750	F03	25281.6667
F04	18743	18979	19153	F04	18958.3333
F05	24293	24328	25192	F05	24604.3333
F06	29319	29764	31553	F06	30212
F07	22469	22836	24319	F07	23208
F08	28734	29284	30916	F08	29644.6667
F09	25925	24738	26099	F09	25587.3333
F10	18342	18499	19502	F10	18781
F11	24816	25357	26719	F11	25630.6667
F12	24747	25619	27103	F12	25823
G01	36282	36544	37897	G01	36907.6667
G02	26597	27705	29607	G02	27969.6667
G03	21605	22251	23368	G03	22408
G04	21789	22862	24249	G04	22966.6667
G05	13290	13525	14153	G05	13656
G06	20218	20942	21981	G06	21047
G07	72504	65619	61343	G07	66488.6667
G08	16012	16474	17007	G08	16497.6667
G09	24241	24729	26300	G09	25090
G10	15017	15366	16003	G10	15462
G11	21981	22321	23385	G11	22562.3333
G12	34877	37085	39109	G12	37023.6667
H01	13778	14703	15742	H01	14741
H02	14180	14921	15698	H02	14933
H03	34520	34179	35130	H03	34609.6667
H04	30349	32687	34127	H04	32387.6667
H05	9127	9729	10026	H05	9627.33333
H06	22155	23621	24712	H06	23496
H07	22173	23054	24328	H07	23185
H08	23743	24991	26213	H08	24982.3333
H09	23394	23769	24982	H09	24048.3333
H10	32818	34415	36500	H10	34577.6667
H11	37137	38193	40497	H11	38609
H12	19066	20078	21413	H12	20185.6667
				average:	23056.7049

Table S4: Measures of the luciferase activity of plate figure 1 with MS2-UPF3X strain.
Reading 2 was performed 2 minutes after reading 1
and reading 3 was performed 2 minutes after reading 2.
Amlexanox position (G07) is highlight
