# Supplementary figures and images for: Rescue of nonsense mutations by amlexanox in human cells
Source: Orphanet J Rare Dis. 2012 Aug 31;7:58. doi: 10.1186/1750-1172-7-58 (PMC3562214; doi:10.1186/1750-1172-7-58)

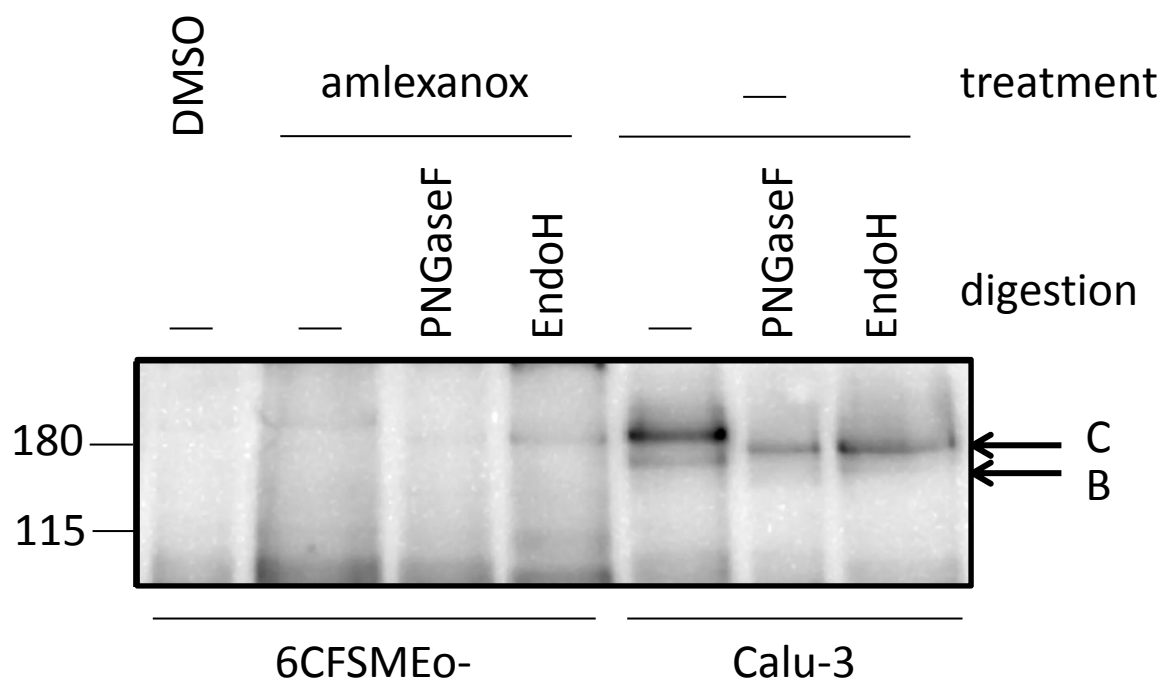

Figure S1

Supplement: Additional file 2 — Figure S1. Analysis of the glycosylation status of CFTR in 6CFSMEo- treated with DMSO or 25 μM of amlexanox for 24 hours and Calu-3 cells. 1/10 of CFTR immunoprecipitation from Calu-3 cells and 1/2 of CFTR immunoprecipitation from 6CFSMEo- cells were analyzed by western-blot. Molecular weight marker is indicated on the left side of the gel. Band B (B) and band C (C) are indicated on the right side of the gel. This analysis is representative of three independent experiments. [file 1750-1172-7-58-S2.pdf]

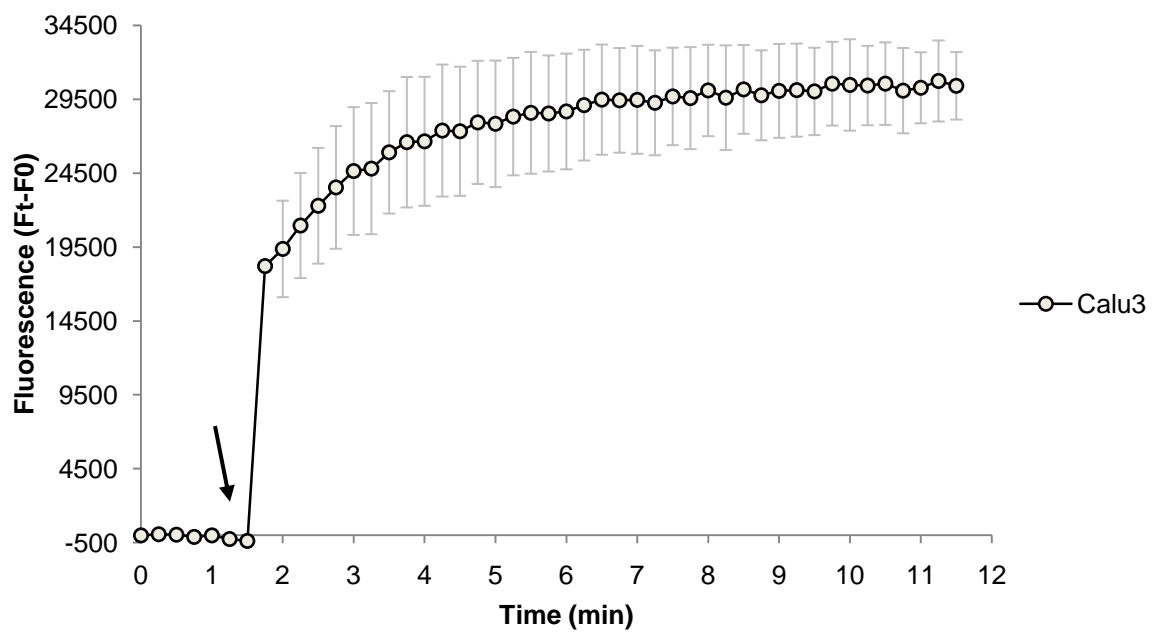

Figure S2

Supplement: Additional file 3 — Figure S2. Measure of iodide transport through Calu-3 cell membrane using halide-sensitive fluorophore SPQ assay. At time (t) = 2 min cAMP-stimulating cocktail was added (arrow). The increase in iodide efflux is shown as mean ± SEM from at least four independent experiments. Ft represents the measure of the fluorescence at the reading time; F0 is an average fluorescence before addition of cAMP agonists. [file 1750-1172-7-58-S3.pdf]
